# A phenomenological model of whole brain dynamics using a network of neural oscillators with power-coupling

**DOI:** 10.1038/s41598-023-43547-3

**Published:** 2023-10-07

**Authors:** Anirban Bandyopadhyay, Sayan Ghosh, Dipayan Biswas, V. Srinivasa Chakravarthy, Raju S. Bapi

**Affiliations:** 1 Indian Institue of Technology Madras, Biotechnology, Chennai, 600036 India; 2 IIIT Hyderabad, Biotechnology, Hyderabad, 500008 India

**Keywords:** Computational biology and bioinformatics, Neuroscience, Mathematics and computing

## Abstract

We present a general, trainable oscillatory neural network as a large-scale model of brain dynamics. The model has a cascade of two stages - an oscillatory stage and a complex-valued feedforward stage - for modelling the relationship between structural connectivity and functional connectivity from neuroimaging data under resting brain conditions. Earlier works of large-scale brain dynamics that used Hopf oscillators used linear coupling of oscillators. A distinctive feature of the proposed model employs a novel form of coupling known as power coupling. Oscillatory networks based on power coupling can accurately model arbitrary multi-dimensional signals. Training the lateral connections in the oscillator layer is done by a modified form of Hebbian learning, whereas a variation of the complex backpropagation algorithm does training in the second stage. The proposed model can not only model the empirical functional connectivity with remarkable accuracy (correlation coefficient between simulated and empirical functional connectivity- 0.99) but also identify default mode network regions. In addition, we also inspected how structural loss in the brain can cause significant aberration in simulated functional connectivity and functional connectivity dynamics; and how it can be restored with optimized model parameters by an in silico perturbational study.

## Introduction

An important question in contemporary neuroimaging research is to understand how the relatively static structural organization of the brain gives rise to brain dynamics and behavior. Structural connectivity (SC) is defined as the physical map of the brain represented by the white fiber tracts connecting different brain regions. SC graph, formed by brain regions as nodes and fiber tracts connecting them as edges, can be represented as a matrix. The elements of the SC matrix are normalized fiber densities across pairs of regions. The resulting SC graph/matrix can be analyzed using measures from graph theory to investigate small worldness and how the pattern of connectivity constrains the information flow and communication between brain regions. On the other hand, functional connectivity (FC) represents the correlation of blood oxygen level-dependent (BOLD) signals between brain regions, typically estimated by the standard Pearson correlation coefficient. In order to drive home the importance of structural connectivity information and its relation to the function of the brain, in 2005, Hagmann, Sporns and others coined the term “connectome”^1,2^.

The structural information extracted from diffusion tensor imaging (DTI) is useful in detecting aberrations in neuroanatomical structures in various neurological disorders. In recent times, resting-state functional MRI (rs-fMRI), recorded when participants are not engaged in any task but are resting, has given valuable insights into the brain function both in health and disease. It has been shown that the low frequency BOLD oscillations in the resting state can be predicted reliably from the structural connectivity information^3^. Networks of regions co-active during rs-fMRI have been designated as resting state networks (RSN), and it was shown that the core of the resting state functional connectivity network corresponds to the nodes of the default mode network (DMN)^4^.

The characterization of the structure-function relation has been attempted using broadly three strategies. According to the first of them, in the case of reaction-diffusion-based models, regional mean firing rates are determined with the help of the popular Wilson-Cowan neuron model, and diffusion of mean firing rate is mediated by the anatomical pathways constrained by the empirical structural connectivity^5,6^. The second broad strategy is to construct biologically plausible neural mass models comprising firing patterns of both excitatory and inhibitory populations of neurons, with parameters that control local and global interactions among the populations^7^. The parameters are estimated by a process of optimization with the objective of matching the simulated FC with the empirical FC^8^. The third kind is nonlinear dynamical system-based models that incorporate abstract phenomenological models of neuron populations based on oscillators such as - the Kuramoto oscillator and the Hopf oscillator^9–12^. These types of models are computationally more tractable but pose challenges of biological plausibility and validity.

Oscillator models offer an appropriate level of trade-off between complexity and biological plausibility for modeling mesoscale data such as resting-state FC and SC. Several example models with Hopf oscillator-based dynamical systems are developed that have yielded promising results in explaining the structure-function relationships, relationships related to cognitive behavior, sleep-wake cycle, Schizophrenia, Alzheimer’s disease, etc^11,13–17^. Models developed by Deco et al. show that a linearly coupled Hopf-oscillator system, where dynamics of every brain region are described by an individual Hopf-oscillator, can explain complex characteristic behaviors of the human brain like connectivity, criticality, information processing, and metastability^11,18^. The model dynamics is varied using several parameters, like - global coupling between different brain regions (G), oscillation amplitude in terms of the bifurcation parameter ($\mu$), and time delay between regions^11,14,19^.

However, one of the major drawbacks of such models is that they cannot accurately capture the empirical BOLD signals from multiple ROIs (Regions of interest). For example, the correlation coefficient value between simulated and empirical FC in these modeling approaches ranges between 0.72 to 0.82^11,20–22^. Approximating the empirical state of a healthy brain in terms of the measure like predictive power (the correlation coefficient between predicted and empirical FC matrix) is necessary for not only achieving the mechanistic understanding of the resting-state human brain but also to understand the brain disorders in the terms of breakdown in the structure-function relationship. Furthermore, such models enable validating the efficacy of neurorehabilitation techniques and suggesting target sites for deep brain stimulation, transcranial magnetic stimulation, etc^21^. One of the dominant approaches in the above described models is to estimate different parameters controlling the dynamics of the system like SC with the scaling parameter (G), the inherent time delay between the brain regions, and the amplitude of the oscillation by a cumbersome process of optimization. Table  1 presents a taxonomy of different models and compares them on several dimensions.

To address these issues, we propose a network of complex Hopf oscillators coupled by a special form of complex coupling called “power coupling”^23^. The dynamics of Hopf oscillators are described in the complex domain, and the complex-valued outputs of the oscillators are passed through a complex feed-forward network which predicts BOLD signals from multiple ROIs. The weight-update rule used in the proposed model poses high biological plausibility and does not resort to an abstract optimization process.

The contributions of the paper are: (i) hierarchical oscillator neural network model architecture where the first layer is constrained by empirical structural connectivity and the last layer predicts functional connectivity among ROIs, thereby modelling the structure-function relationship, (ii) model estimation using a variation of complex backpropagation algorithm, (iii) modelling static as well as dynamic functional connectivity, (iv) comparison of the graph theoretical properties of the predicted FC with empirical FC, and (v) extensive perturbation studies, including a study of the impact of the loss of structural information and the impact of various parameters in restoring the aberration in function.

This paper is organized into four parts - after the first introductory section, the results section describes the outcomes of various simulation studies; the discussion section outlines the similarities and differences of the proposed model in comparison to the earlier models, and highlights the superior predictive power of the proposed model; and finally, the methods section describes the learning algorithm and in-silico perturbation studies.

## Results

### Model abstract

In this paper, we propose a general trainable oscillatory neural network for modelling the relation between FC and SC. The proposed network uses Hopf oscillators whose dynamics are described in the complex domain. A distinctive feature of the model is the manner of coupling among the Hopf oscillators. Instead of the linear coupling between two oscillators explored in earlier works, we use a form of coupling known as power coupling. Oscillatory networks based on power coupling have been shown to be able to learn arbitrary multi-dimensional signals^23^. The network consists of a layer of oscillators, followed by a layer of sigmoidal neurons, and finally, the output layer that captures the BOLD activity of various ROIs. Training is done by a variation of a complex backpropagation algorithm^24,25^, which can train the hybrid network of oscillators and sigmoid neurons. The objective of the training is to find the best fit between the empirical and simulated BOLD signal for individual ROIs. The proposed model not only emulates the empirical FC, but also investigates if the model can simulate the dynamic FC and matches graph theoretical measures. A more detailed account of model architecture and function is presented in the Methodology section.

### Reconstruction of the BOLD signal with novel network architecture

**Figure 1:**
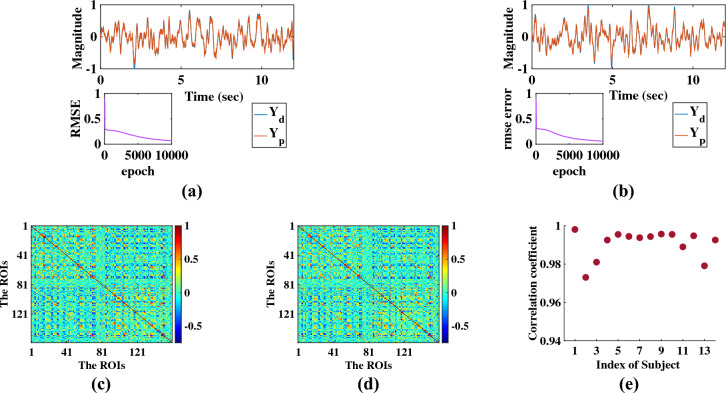
(**a**) and (**b**) represent the comparison between simulated and empirical time-series signals for ROI-1 and ROI-2, respectively. $Y_{d}$ and $Y_{p}$ represent empirical and simulated signals, respectively. (**c**) and (**d**) represent the “grand-average” simulated and empirical FC respectively of fourteen participants. (**e**) shows the individual correlation coefficient between simulated and empirical FC for each of the fourteen participants from the HCP dataset.

Figure 1a and b reveal that after the simulation, the reconstruction of the BOLD signals matches the empirical ones. Each simulation is done for 10,000 epochs, and the number of hidden nodes for each ROI is fixed at 30. Note that only two ROI’s simulated and empirical BOLD signals for the second participant from HCP dataset are shown in Fig. 1. Like the first two ROIs, all the other empirical BOLD signals from 160 parcellated regions can be reconstructed with this model for all the participants.

The effect of various parameters on the performance of the network has already been described earlier^25^. It has been found that the network-performance is a function of both the number of hidden neurons and the number of epochs, where the correlation coefficient between empirical and simulated FC increases monotonically with the number of epochs and the number of hidden neurons. First, the Human Connectome Project (HCP) dataset is used to reconstruct the BOLD signal extracted from the total of 160 ROIs. Note that the model keeps the individuality of the structural connectivity of each participant, preserving the “structural biomarker” of each subject, and that is the reason why the model architecture is different for different subjects.

### Functional connectivity, and functional connectivity dynamics

**Figure 2:**
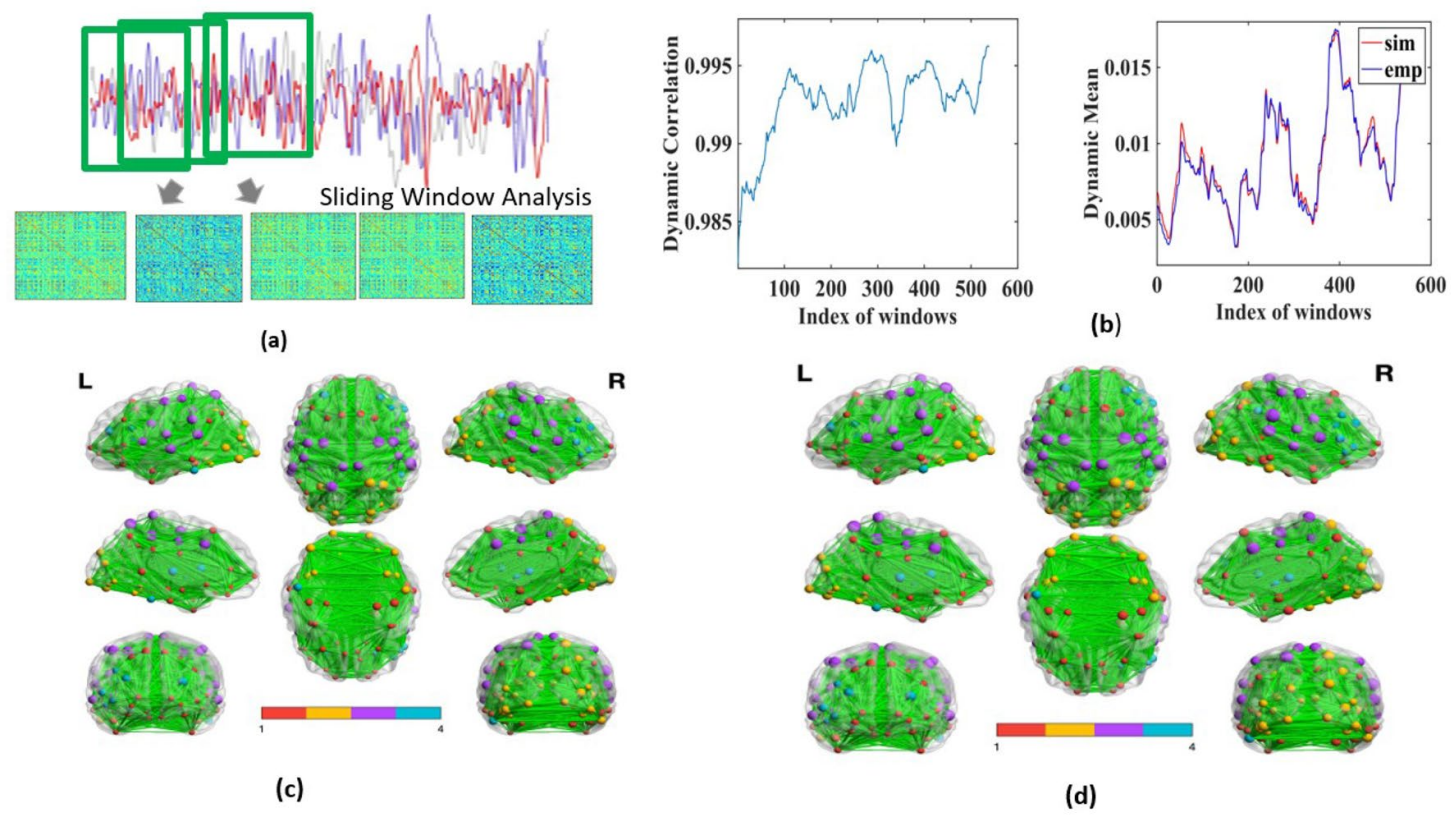
Functional connectivity dynamics (FCD) with sliding window analysis(SWA). (**a**) in the upper panel shows the procedure of sliding window analysis, and (**c**) and (**d**) in the lower part show the ROIs as nodes of the brain network. (**b**) shows that dynamic correlation between simulated and empirical FC; and the dynamic mean of functional connectivity for each sliding window. The lower panel in (**c**) and (**d**) refer to the virtual brain view to identify different communities marked by individual colour, where the node’s size corresponds to the node’s degree. This is developed using Brainnet Viewer^26^.

The FC Matrix presents itself as a powerful tool for revealing the brain dynamics underlying fMRI data, classifying brain networks, and extracting the hierarchical organization in such networks^27,28^. The novel network architecture can also emulate significant biomarkers like FC and functional connectivity dynamics (FCD). Analyzing the FC matrix and comparing simulated and empirical FC is a promising benchmark for inspecting a model’s capacity^11,21,29,30^. The correlation coefficient between the simulated and empirical FC matrix approximates 0.99 (from the first participant’s data), accurately capturing the empirical FC matrix. A set of fourteen simulations for fourteen subjects are performed, and the “grand average” empirical FC and simulated FC are shown in Fig. 1c and d , and the individual correlation coefficient between the simulated and empirical FC matrix is displayed in Fig.  1e.

Several earlier works postulate that understanding the static FC matrix may not be the ideal approach to validate the model^11,12^. FCD is investigated with the procedure outlined in the Methodology section. How the dynamic functional connectivity changes with sliding windows is shown in Fig. 2a. Figure  2b shows how the correlation coefficient measuring the model’s performance on dynamical nature of empirical FC, alters with the shifting window, which ranges between 0.980 to 0.995, and reveals the dynamic mean of FCs between simulated and empirical BOLD signals. This shows the dynamic nature of FC can be captured by the model. Note that the results shown here are derived from the simulated and empirical BOLD signal output from the first participant’s data. Results for the “brain-states” are divulged in Section S3 and Fig. S2 in supporting documents online. Four different FC matrices, as described by “brain states”, are concatenated as per the procedure outlined in the Methodology section. The Pearson correlation coefficient between two large FCD matrices is estimated to be 0.99.

### Brain graph theory, and default mode network

As described in the Methodology section, Newman’s community detection algorithm is chosen for performing the segregation and analysis of the default mode network (DMN). The modularity (Q) value for the network derived from empirical data is estimated to be 0.33, and for the simulated network, it is 0.34. Four modular structures are found in both the empirical and simulated graph, as shown in Fig.  2c and d; and also in Fig. S3 in Section S4 in the supporting document online. The posterior cingulate cortex is a well-known region in DMN, positioned in the 9*th* and 10*th* index in the parcellation table given in Table S2 in the supplementary document online, segmented in cluster 1^31,32^. It is found that some important regions of DMN are in the similar community, like- the anterior cingulate cortex, posterior cingular cortex, medial prefrontal cortex, angular gyrus, parahippocampal regions, Precuneus, superior frontal gyrus, superior temporal gyrus, middle temporal gyrus, temporal pole, temporal inferior gyrus, hippocampus^33–37^. However, some of the ROIs are also present in the community, which are not typically regarded as a part of the DMN network.

### Effect of information loss and recovery

The dysfunction of structural connectivity in the brain is observed in various neurological disorders^38,39^. The current section describes the results of in silico perturbation and rehabilitation studies. The simulations are done on different SC matrices, which are randomly pruned, resulting in anomalous FC matrices. The next objective is to retrieve the FC matrix prior to the structural pruning process by altering the different parameters governing the Hopf oscillator dynamics. Note that the pruning process is not a targeted attack but a random one. Both targeted and random attack on SC and their effects on simulated FC are reviewed in earlier works^38,40^.

**Figure 3:**
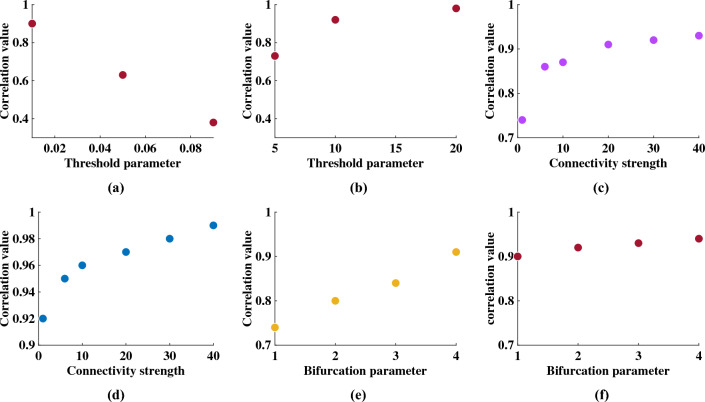
How the correlation coefficient between simulated and empirical static FC varies with several model parameters is shown here. The threshold parameter shown in (**a**) is the absolute threshold. In (**b**), the threshold parameter indicates the percentile of the stronger weights in the SC matrix, which are retained after pruning. Both (**c**) and (**d**) show how the global coupling strength (G) can restore the correlation coefficient between simulated and empirical FC matrix, when five, and ten percentile threshold on SC is applied, respectively. (**e**) and (**f**) show how the increase in oscillation amplitude $\mu$, can compensate for the information loss due to structural lesions incurred due to five, and ten -percentile threshold.

After the absolute thresholding process, as shown in Fig. 3a, the correlation value between simulated and empirical FC (predictive power) decreases monotonically with the increasing threshold. Similarly, in the case of proportional thresholding, where a certain percentile of stronger connections are retained, the predictive power increases with the percentile of stronger weights (Fig. 3b). The correlation coefficient between simulated and empirical FC is lower for five percentile retained weights than for twenty percentile retained weights. We can compare our oscillatory model with the temporal kernel model developed by Surampudi et al.^5^, where a similar pruning exercise was done.

**Figure 4:**
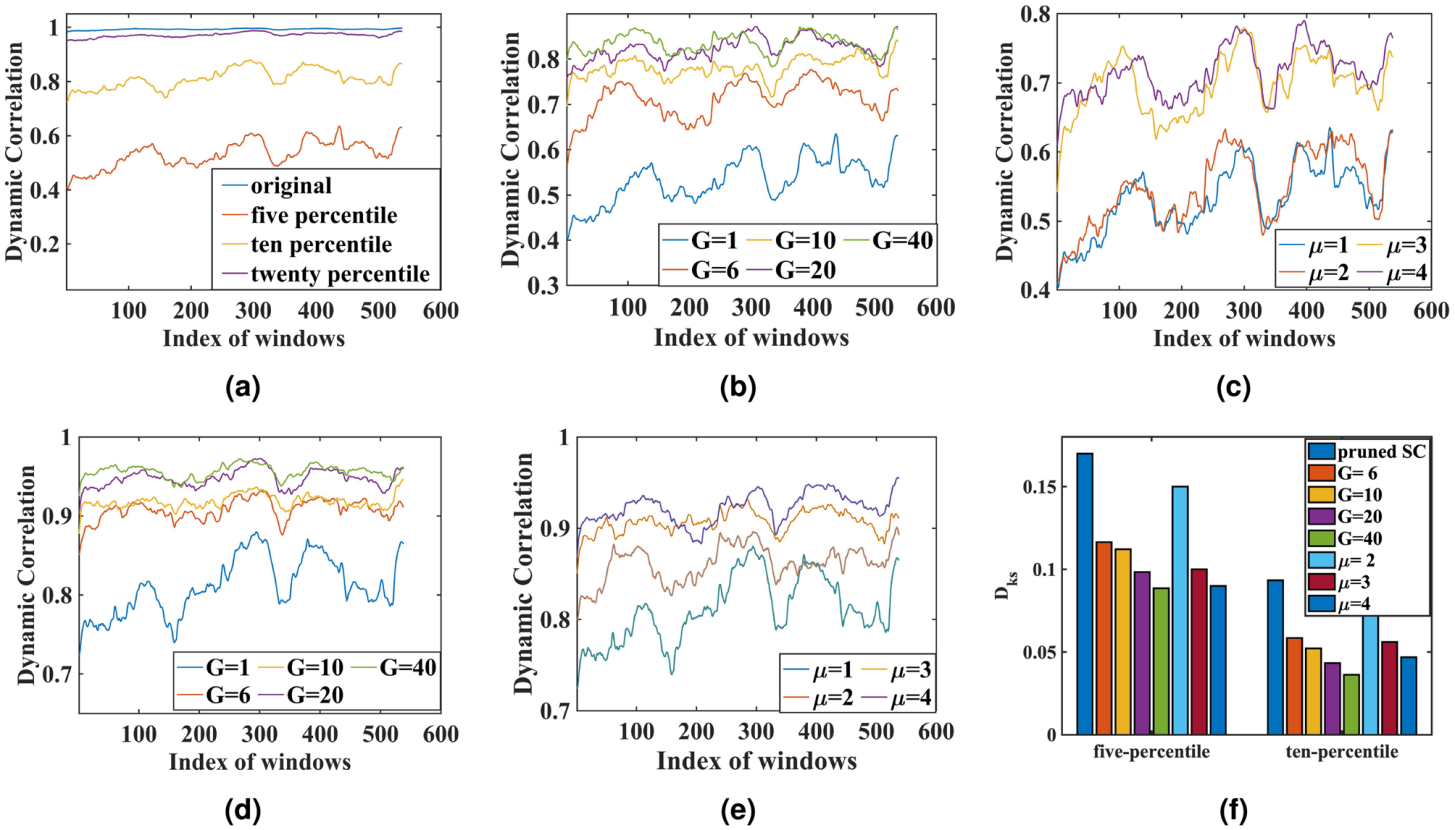
(**a**) denotes the dynamic correlation in case of no proportional threshold (referred to as ’original’), five-percentile threshold, ten-percentile threshold, and twenty-percentile threshold. (**b**) refers to the dynamic correlation, when G is increased monotonically for five percentile pruned SC. (**c**) refers to when $\mu$ is increased from $\mu = 2$ to 4; the dynamic correlation value is increased in the case of SC matrix with five percentile threshold; (**d**) reveals the FCD in the case of ten percentile threshold, where increased G is used. (**e**) depicts the alteration in dynamic correlation for various values of the oscillation amplitude $\mu$, and when the SC matrix is pruned with ten percentile threshold. (**f**) reveals the Kolmogorov-Smirnov($D_{ks}$) distance to underpin the difference in dynamic correlation distribution between before and after perturbation (pruned SC and pruned SC with regulatory parameters).

In the rehabilitation process, it is intended to restore the FC, which is imbalanced due to structural loss. In order to achieve this, as discussed in the Methodology section, the dynamics of the Hopf oscillators are altered, where the SC matrix is scaled with global coupling parameter G; and the amplitude of the periodic oscillation of the Hopf oscillator ($\mu$) is increased. Note that only proportional threshold is taken for this in silico rehabilitation study. It is noted that with the increase of G, the predictive power or the correlation coefficient increases monotonically. The results are shown in Fig. 3c and d, where the analysis of restoring the predictive power is performed on two different pruned structural connectivity data sets – five percentile, and the ten percentile proportional threshold applied to the SC matrix. Note that the first point in both cases is the initial point where the G is kept at 1. It should be noted that $\mu$ is kept constant in this case. It is also found that when $\mu$ is increased, the model’s predictive power increases; however, G is kept constant in this case, as shown in Fig. 3e and f , revealing that restoration of the model’s predictive power or correlation coefficient is possible. $\mu$ is increased up to $\mu$= 4 for the five percentile, and the ten percentile proportional threshold applied to the SC matrix as shown in Fig. 3e and f. It is to be noted that the nodes are assumed to be homogeneous in nature, meaning all the Hopf oscillators have the same amplitude of oscillation, $\mu$. A detailed description of the impact of structural loss and its possible compensation is given in Section S5 of the supporting document online. In the future, we intend to study node heterogeneity to inspect how the node parameters are crucial for fitting the model to clinical data. For example, increased amplitude of oscillation can be linked to invasive and non-invasive brain stimulation methods, deep-brain stimulation (DBS), Transcranial Direction Current Stimulation (tDCS), and Transcranial Magnetic Stimulation (TMS). One instance of such an effort is the study by Iravani et al.^21^, where the model accounts for the effects of focal stimulation in ADHD participants. Model’s performance on FCD analysis, for in silico perturbational study, is done in terms of dynamic correlation, where each element denotes the Pearson’s correlation coefficient between the upper-triangular elements of the simulated and empirical FC matrix at individual time-window defined by the sliding window analysis. A similar strategy is applied to inspect the impact of structural loss and recovery on FCD analysis, as shown in Fig. 4, where the variation of correlation with time is given for different pruning conditions and the subsequent recovery process, as outlined in the "Methodology" section. Figure 4a shows the dynamic correlation obtained from structural connectivity without threshold, and structural connectivity with five, ten, and twenty percentile threshold values. Figure  4b–e reveal the impact of regulatory parameters, G, and $\mu$ to restore FCD after perturbation to the prior pruning stage. A closer look will reveal that the ranges of dynamic correlation increase with the increase of the regulatory parameters. It will be more conspicuous from Fig. 4f, where the dynamic correlation obtained from different perturbation and rehabilitation strategies, as outlined earlier, is compared with the dynamic correlation obtained from SC without pruning (Fig. 2b) with the help of the Kolmogorov-Smirnov distance ($D_{ks}$). In Fig. S12 in the online document, we have shown the distribution of dynamic correlation value for each perturbation condition. It should be noted that, though the result shown here is for a single participant, the general trend is true for all other participants.

### Validation dataset

We now check if our model can capture the whole brain dynamics in a different dataset, other than the HCP dataset, having a different repetition time (3.29 seconds) with nearly 200 time points.

**Figure 5:**
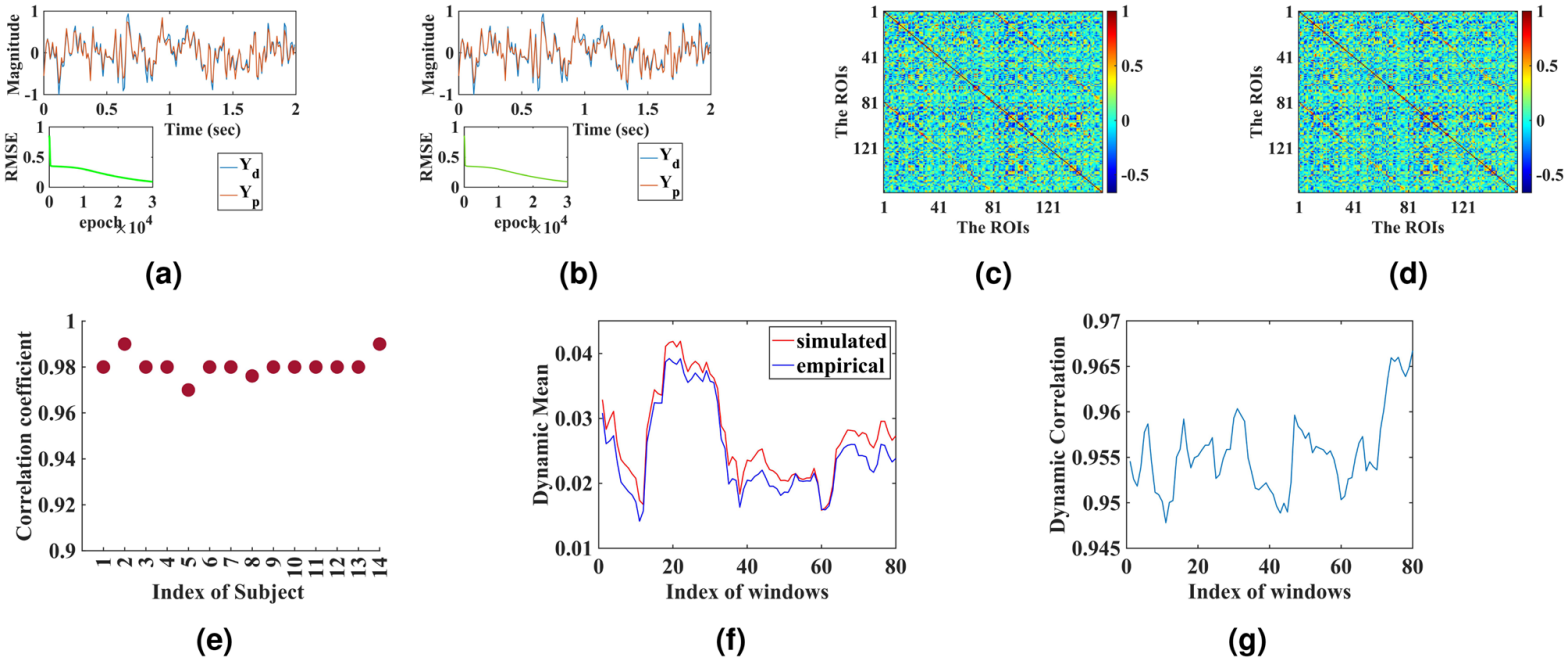
Simulated Outcome for validation dataset. (**a**) and (**b**) individually represent the first and second ROI’s BOLD signal approximation with our model. (**c**) and (**d**) reveal the simulated and experimental functional connectivity; (**e**) presents the predictive power of the model for the fourteen participants of Paris dataset. (**f**) and (**g**) represents the dynamic functional connectivity for the first participant of the Paris dataset.

As in the case of previous analysis with the HCP dataset, our model can reconstruct the BOLD signal extracted from 160 ROIs of each participant from the Paris dataset. The architecture is kept constant, and the model architecture is identical to the one used for the HCP dataset. The simulated results after 30,000 epochs with thirty hidden neurons in the hidden layer are shown in Fig. 5a and b. As per the methods developed earlier, the capacity of the model is described in terms of the correlation coefficient between the empirical and the simulated FC (see 5c and d). The correlation coefficient value after 30,000 epochs with 30 hidden neurons is estimated to be 0.97 for the first indexed participant from the Paris dataset. Figure 5e reveals the correlation coefficient between simulated and empirical functional connectivity between all the fourteen participants from the Paris dataset. Note that the window size for FCD analysis is kept at 40 TR. Both the mean FC and the dynamics correlation coefficient across the windows are estimated, as shown in Fig. 5f and g.

## Discussion

Recent reviews have highlighted the importance of building large-scale brain models, their significance to brain theory, and their applications in clinical neuroscience^10,12,30^. Large-scale models validated by whole brain BOLD signals present a theoretical framework which can be used to predict the underlying neurophysiological events, to serve as a platform for developing effective neuro-rehabilitation techniques. Prior works in such direction show promising results that can relate the parameters in model dynamics with the external perturbations, and normal and disease conditions. Vattikota et al. showed in their work that a virtual focal lesion can be rehabilitated by a re-balancing of the excitatory and inhibitory dynamics^40^. Such a perturbation study is also done in our work to check whether our model can also probe into such information of perturbation transferred from SC to FC, and whether any local and global level alteration can cause any difference in the FC reconstruction after perturbation. Here the increase of oscillation amplitude ($\mu$) signifies local level alteration, and the pruning of structural connectivity, or its scaling using global coupling factor (G) refers to global level perturbation. However, in the current work significance of the oscillatory power analysis is restricted, where the amplitude of oscillation is increased homogeneously for all nodes instead of selective one. Note that a change in the number of connections, or connection properties or the number of neurons, or neural activity, in selective areas of the connectome is a dominant pattern in neurological disorders, like “disconnection” in Schizophrenia, stroke, Parkinson’s disease etc^39,41,42^. It has been observed that such perturbations can sometimes be restored by either a self-reorganization of the structural connectivity or by modulation of synaptic plasticity^43–45^. Our work shows that the increase in the global coupling factor (G) can ameliorate the anomalous FC derived from the disconnection in SC. Prior simulation studies of the effect of deep brain stimulation (DBS) in Parkinson’s Disease patients reveal that by varying the single global coupling factor, G, it is possible to estimate global segregation and integration measures in pre-DBS, post-DBS, and healthy conditions^13^. Work done by Cabral et al. shows that by lowering the G, the dynamic system model can match the global integration (GI) value derived from the empirical BOLD signals extracted from patients suffering from Schizophrenia diseases^45^. On the other hand, the importance of the increase in oscillation amplitude is apparent in DBS intervention in Parkinson’s disease, where the thalamus and global pallidus may be associated with a higher amplitude of oscillation^46^. Another work done by Iravani et al. shows the implication of an increase in the amplitude of oscillation resulting from the increase in $\mu$ shows promising results for Attention-deficit/hyperactivity disorder (ADHD) subgroup-1 patients, where the left-medial orbitofrontal cortex and right posterior cingulate cortex are found to be the most promising sites for focal brain stimulation^21^. In an in silico study (Deco et al. 2018), the perturbational landscape of the linearly coupled Hopf oscillator model of Deco et al.’s points out the impact of $\mu$, in terms of periodic and noisy behaviour of Hopf oscillator, in synchronization^47^. These works substantiate the importance of the Global coupling factor (G), and the oscillation amplitude ($\mu$) in modeling clinical conditions, which is reinforced by the results obtained from the proposed model.

**Figure 6:**
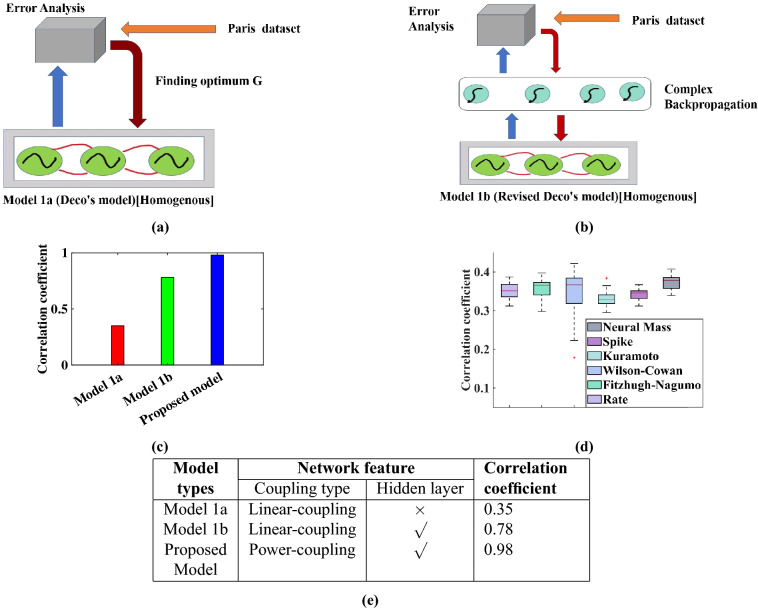
Comparison between different models. (**a**), and (**b**) refer to different models developed based on the linearly coupled Hopf-oscillator model developed by Deco et al. (2017), as shown in (**a**), and an induced hidden layer with a complex back-propagation rule where the first layer is a linear-coupled Hopf-oscillator system which is shown in (**b**). (**c**) indicates the correlation value between simulated, and empirical FC (for first participants’ data) deduced from the three different types of models as described in (**e**), which deciphers the contrasts and performance of different models. (**d**) refers to the comparison of our model with other models. The result (for fourteen participants’ data) is reproduced from earlier work submitted in online repository^48,49^.

We now discuss the proposed model and compare it with previous models that have been proposed to work out the mapping between structural and functional connectivity^15^. As discussed earlier, the characteristic behavior of the Hopf oscillator in the supercritical regime renders a powerful tool for understanding the underlying dynamics of BOLD signals elicited by the different brain regions captured with imaging techniques, like rs-fMRI. Several earlier studies have explored the effectiveness of the Hopf oscillator in elucidating the temporal dynamics of BOLD signals as well as whole brain dynamics. The model proposed by Deco et al. (2017), also based on Hopf oscillators, relies on a linear coupling strategy between oscillators, and the optimal fit to the brain dynamics was found near the bifurcation point ($\mu \approx 0$), determined by the maximum correlation coefficient between the simulated and empirical FC matrix^11^. In subsequent studies, Deco et al. (2017, 2021) computed the intrinsic frequency of the oscillator based on the strongest frequency component of the empirical BOLD signal, and $\mu$ is optimized based on the strength of that frequency component^50,51^. Later this model was also optimized with swarm particle parameter optimization technique^14^. A similar nature of model was described by Iravani et al.(2021), where a similar Hopf oscillator-based model is employed; however, Monte-Carlo optimization process was applied for parameter optimization, and it was found that the parameter distribution can discriminate between the healthy and ADHD brain^21^.

**Table 1:** Discussion between Different Models with the current model^5,11,20,21^.

Model	Hopf-oscillator model	Structural connectivity	Correlation coefficient (approx)	Model description	Clinical importance
Model 1^11^	$\surd$	$\surd$	0.75	Structural Connectivity dependent Hopf oscillator model with linear coupling system	Default mode network identification
Model 2^5^	$\times$	$\surd$	0.80	Multiple kernel learning model with modified Wilson-Cowan based neuron activation	Mapping from FC to SC, and inspecting FC aberration due to structural loss
Model 3^21^	$\surd$	$\surd$	0.82	Hopf oscillator based model with parameter optimization with Monte-Carlo simulation	Difference in parameter distribution of ADHD and healthy brain, and target location for TMS
Model 4^20^	$\surd$	$\surd$	0.82	Hopf scillator based model with rigorous parameter optimization	Analysis of sleep, awake, and anasthesia condition of healthy brain in human and monkey
Current model	$\surd$	$\surd$	0.99	Fourier like decomposition, and retrieved with oscillatory neural network with complex backpropagation strategy	Structural, and functional loss along with possible rehabilitation underpinned by model parameter

The strength and appeal of the proposed model lie in the nonlinear coupling mechanism adopted, viz., power-coupling, the Hebb-like learning of lateral connection and the complex backpropagation algorithm for training the forward connections. A comparison between the earlier models and the proposed model is given in Table  1. Note that the models shown in the table are performed on different datasets. A comparison between different whole-brain models with the aforementioned Paris dataset has already been done, which allows us to compare our model with the earlier models^48^. A total of six models are compared, viz. Rate coded model, Wilson-Cowen model, neural mass model, phase oscillator model (Kuramoto oscillator), spike attractor model (dynamic mean field model), and Fitzhugh- Nugmo model. The correlation coefficient between the simulated and empirical FC matrix for all the competing models is shown in Fig. 6d, which demonstrates that our model significantly improves the predictive power and approximately emulates the empirical functional connectivity. For details of those models, readers may refer to the work of Messe et al.^48^. The proposed model is similar to the model proposed by Deco et al.^11^ as mentioned earlier. The major difference is that in the model of Deco et al. (2017), the Hopf-oscillators are coupled via linear coupling, where the global coupling factor (G) is iteratively selected in order to achieve the best approximation with empirical FC. In order to compare the proposed modeling approach with that of Deco et al. (2017), we took two models based on different network paradigms as follows. The characteristic features of the networks are given in the table in Fig. 6e. As shown in Fig. 6a, the model of Deco et al. is employed for Paris dataset to get the simulated output, where the best simulated outcome is found at G = 2.6, and $\mu$ is set to near zero. To make it comparable, the nodes are assumed to be homogeneous in nature, and the $\mu$ is not updated. In Fig. 6b, the oscillatory layer is similar to Deco et al.’s model, and the complex-signal output from the first oscillatory layer is transferred to a sigmoidal neuron layer, and the lateral connections of the oscillatory layer are constrained by the SC matrix as in the second phase of learning model of our model. Figure 6c reveals the correlation coefficient (predictive power) between simulated functional connectivity and empirical one for different network paradigms. This outcome vindicates the implication of both the hidden layer and the power-coupled oscillator system.

In future development, we propose to extend the current model to simultaneously model EEG and fMRI signal, by representing each ROI not by a single oscillator but by a cluster of oscillators. This cluster will have a high frequency subcluster which will primarily model EEG data and a low frequency subcluster which will model fMRI data. In such a model, hidden coupling features between high-frequency bands and low frequency bands are expected to be reflected in the lateral connections between the two subclusters.

## Methodology

### Databases used

We have taken the structural and functional MRI data from online repositories. Two sets of pre-processed fMRI data are used - the popular HCP dataset^52^ , and the Paris dataset^49^. The HCP dataset has forty participants and a total of 4896 (58 minutes) time points with TR (Time of repetition) = 0.72 seconds. We have taken only the first session data for the first fourteen subjects. Subsequently, we have taken the Paris dataset with TR (Time of repetition) = 3.29 seconds for validation. The first fourteen participants are taken for the simulation. The parcellation is done using FreeSurfer software; the eighty parcellated regions for the left hemisphere are listed in Table S1 in online supplementary documents. For a more detailed description of the data extraction process, the readers are referred to Marrelec et al.^49^.

### Basic mathematical model

The proposed network model consists of two stages: (1) an oscillatory stage and (2) a feedforward stage. The oscillatory stage consists of a fully connected network of Hopf oscillators. The dynamics of a single Hopf oscillator can be described in terms of two real variables in cartesian form (*x* and *y*), in polar form (r, $\phi$) or in complex form ($Z = x + iy$). We use the complex form because, in this model, the oscillators are coupled using power coupling^23^, which is more elegantly expressed in complex form. The dynamics of a single Hopf oscillator in the complex domain may be expressed as:^53^—

1 $$\begin{aligned} \dot{Z}= Z(\mu + i\omega -|Z|^2) \qquad in\, polar\, form\, ({r, \phi }), \qquad \dot{r} = \mu r - r^3; {{\dot{\phi }}}= \omega ; \end{aligned}$$

The second stage, which is a feedforward network, consists of a complex-valued multilayer perceptron with a single hidden layer. The outputs of the oscillator layer are presented as inputs to the feedforward network. The entire model consisting of the two stages is trained such that the output of the feedforward network approximates the fMRI data. The dynamics of the network of Hopf oscillators in the supercritical regime, which constitutes the oscillatory stage, are described below –

2 $$\begin{aligned} \dot{Z}_{i}= Z_{i}(\mu + i\omega _{i} -|Z_{i}|^2)+G\sum _{j=1, j\ne i}^{N} A_{ij}e^{i\frac{\theta _{ij}}{\omega _{j}}}Z_{j}^\frac{\omega _{i}}{\omega _{j}} \end{aligned},$$

where, $Z_{i}$ is the complex state of $i{th}$ Hopf oscillator which is in limit-cycle oscillation, $\mu$ is the bifurcation parameter, and G is the global coupling factor, a scalar constant. $W_{ij}$ ($A_{ij}e^{i\frac{\theta _{ij}}{\omega _{j}}}$) is the complex coupling coefficient between $i{th}$ and $j{th}$ oscillator. Note that the coupling term in Eq.  (2) above involves raising $Z_{j}$ to the real power given by $\omega _{i}/\omega _{j}$. This form of coupling is known as “power coupling” and has useful properties discussed in our earlier work^23^. However, A brief summary is given as follows for the convenience of the readers. One of the major concerns about a Hopf oscillator-based coupled system is that of phase synchronization. Several types of coupling strategy between a set of Hopf oscillators have been proposed earlier, like real-valued coupling and complex coupling^11,54^. In these schemes, synchronization is enabled by maintaining constant phase difference. In the earlier models, constant phase difference is achieved when intrinsic frequency of the coupled oscillators is identical. Identical intrinsic frequency is an unreasonable and unrealistic constraint. To address this, in our recent work, we proposed normalized phase difference between two or more Hopf oscillators using power coupling. In the case of power coupling, it has been shown that irrespective of the difference in intrinsic frequencies of Hopf oscillators, the normalized phase difference attains a steady state value and produces a unique phase locked system^23^.

**Figure 7:**
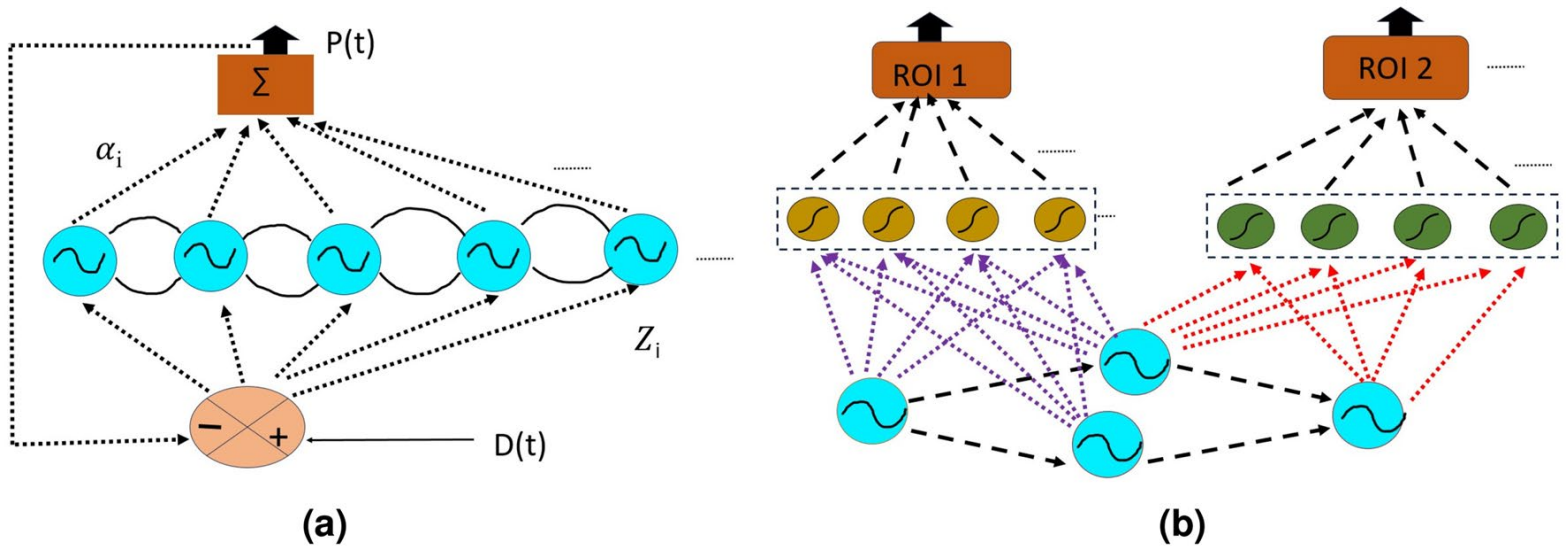
Schematic diagram of the model. (**a**) depicts the network architecture used in the $1{st}$ stage of learning. It consists of the oscillator layer and a linear stage connecting the oscillators with the output layer. (**b**) shows the proposed feedforward network model architecture for $2{nd}$ stage of learning involving one hidden layer consisting of 30 hidden neurons. Figures are adapted from our previous study^23,25^.

In the whole brain model, every Hopf oscillator is positioned at the parcellated Brain regions or region of interests (ROI), acting as a node. The nodes are assumed to be homogeneous meaning parameters like oscillation amplitude, $\mu$, which is kept constant for all the nodes. The second stage of the network, the feedforward network, is used in two alternative forms, depending on the stage of learning. The $1{st}$ stage of learning is a single linear stage, or a perceptron, with complex weights. In the $2{nd}$ stage of learning, it is a complex-valued multilayer perceptron with a single hidden layer (Fig. 7). End-to-end training of the entire network is described below.

#### First stage of learning

The objective of the first stage of learning is to train the oscillatory stage, which consists of training the two sets of parameters of the oscillatory stage, viz, the intrinsic frequencies, $\omega _{i}$, and the coupling weights $W_{ij}$. To this end, we use the training scheme depicted in Fig.  7a. To train the oscillatory stage, we must cast the learning in a supervised learning scheme. In Fig.  7a, we train the network such that a weighted sum of the oscillators approximates a desired signal. Therefore, in this stage of learning, the feedforward stage comprises of the linear stage. There is only one output signal in this stage of learning, which can be the fMRI time-series signal from any one of the ROIs. The primary goal is to extract the frequency components of the ROI signals, with the implicit assumption that the spectral components of all the ROI signals are nearly the same. The learning rule for updating the frequencies, $\omega _{i}$, is followed as Eq.  (3), where *e*(*t*) is the error between the empirical signal, *D*(*t*), and the simulated signal, *P*(*t*)^23,25^.

3 $$\begin{aligned} {{\dot{\omega }}}_{i}= \beta _{w}e(t)\sin \phi _{i} \quad \textrm{where,}\quad e(t)= D(t)- P(t) \quad \textrm{and,}\quad P(t)= \sum _{i=1}^{N}\alpha _{i}\cos \phi _{i} \end{aligned}.$$

The lateral connections, which are complex in nature, $W_{ij}$ are partly trained and partly predetermined using experimental data. While the magnitude, $A_{ij}$, of the lateral connections is set using structural connectivity information, the angle ($\theta _{ij}$) of the lateral connections are trained using a Hebb-like learning rule shown in Eq.  (4) below.

4 $$\begin{aligned} \tau _{w}\dot{W}_{ij}= -W_{ij}+Z_{i}Z_{j}^{{*}{\frac{\omega _{i}}{\omega _{j}}}} \quad \textrm{where,}\quad W_{ij}= A_{ij}e^{i\theta _{ij}/\omega _{j}} \end{aligned}.$$

The values of learning rates like—$\beta _{\omega }$, $\tau _{w}$ are set as $10^4$, and $10^{-4}$. Since the goal of this stage of learning is to train the oscillatory stage alone, after this stage, the linear feedforward weights are discarded, and replaced by a multilayer perceptron which is trained in the second stage.

#### Second stage of learning

In the second stage of learning, we take the trained parameters of the oscillator layer from the previous stage. The linear feedforward network, used earlier, is replaced by a complex-valued multilayer perceptron with a single hidden layer^24,25^. However, unlike the previous stage, wherein all the oscillators are connected to all the output neurons, in this stage of learning, oscillators are coupled to the neurons in the hidden layer more selectively. Note the SC network is not a fully connected network; it is a sparse one since every brain region or ROI is not connected to every other ROI in the brain. Therefore, each ROI is associated with a separate network which consists of a single (say, $i{th}$) oscillator, a single output neuron, the $i{th}$ output neuron, and a separate hidden layer of size K, mediating between the two. The oscillator corresponding to a given ROI is connected to all the K neurons in the associated hidden layer. In addition, all the oscillators to which the $i{th}$ oscillator is connected by non-zero structural connectivity rendered by the empirical SC matrix, also project the same set of K hidden neurons corresponding to the $i {th}$ ROI (see Fig.  7b).

Note that, only real value of the network output is taken as a simulated BOLD signal, where the imaginary part represents the hidden state of the scanner.The governing equations for constructing the network are given in Section S1B in the supplementary document online. Also the parameters associated with the model has been provided in Section S1 in the supplementary document online. For more information about the network hyper-parameters (number of epochs and hidden neurons) as a function of network efficiency, readers can refer to our previous work^25^.

### Analysis of fMRI signal- FC, FCD

The element at (*i*, *j*) in FC is basically the Pearson Correlation Coefficient between the signals from the $i{th}$ and $j{th}$ ROIs. FC can be viewed as an adjacency matrix, where different brain regions act as nodes, and the edges are the correlation coefficients. Functional connectivity dynamics analysis (FCD) is done for analyzing both simulated and empirical BOLD signals^55–57^.

However, there is no singular, universally accepted protocol to perform FCD. For FCD analysis, the following steps are taken : (1) For the sliding window analysis (SWA), window size and slide length are kept constant at 120 TR (83.60 seconds) and 2TR (1.44 seconds). (2) A total of 538 windows are found. For estimating dynamic correlation, the correlation coefficient between $m{th}$ simulated and empirical FC is calculated individually for $m{th}$ sliding window, and the dynamic mean at $m{th}$ position is computed by taking the mean of upper-triangular elements of $m{th}$ simulated and empirical FC. Another way of understanding the FCD can be done by “brain state” analysis, where the protocol outlined by Menon et al.^58^ is adopted. The steps are as follows: (1) K -means clustering algorithm is used to classify the FCs derived from SWA into different states, and the centroids of the states are identified. (2) The states are sorted according to the number of elements in each cluster in ascending order to avoid the problem of random initialization in the K- means algorithm in MATLAB. (3) Finally, all the states are concatenated, and the distance between them in terms of the correlation coefficient is noted. The same process is repeated for both simulated and the empirical fMRI signals.

### Brain graph analysis, and default mode network

The theoretical measures of the brain network can be computed both with simulated and empirical FC. Recent advances in the brain-graph theory reveals that the brain works in a highly modular but integrated fashion; where some ROIs are clustered with high connectivity among themselves, and low connectivity with others. We use modularity (Q) as a quality function for segregating different communities^59^. The modularity (Q) can be defined by -

5 $$\begin{aligned} \begin{aligned} Q (G,S) = 1/4m \sum _{ij}\left[A_{ij}-\frac{K_iK_j}{2m}\right]S_iS_j \end{aligned} \end{aligned},$$

where, $A_{ij}$ is the adjacency matrix, $\frac{K_iK_j}{2m}$ represents the expected number of edges within group S. $K_{i}$, and $K_{j}$ are the degrees of the nodes. $S_i$ represents if the current node is in the community or not.

The procedure for identifying DMN nodes is given below- (1) The adjacency matrix is created from the “grand average” simulated and empirical FC. (2) The self-connections and the negative connections are set to zero. (3) Later, Newman’s community detection algorithm is used to identify the cluster or module (set of highly connected nodes) with the help of the modularity score, determining the segregation level in the network. The MATLAB code for the algorithm is already available in the Brain connectivity toolbox^27^. And finally, the community affiliation vector and the corresponding nodes in the specific community are noted to determine the ROIs belonging to DMN regions.

### Perturbation study and rehabilitation strategy

Structural damage or a lesion is a common underlying pathology in neurological disorders like stroke, Parkinson’s, and Alzheimer’s disease^41,60^. Here an in silico study is done to determine the effect of loss of structural information on functional information in terms of correlation coefficient (predictive power) estimated by the correlation coefficient between simulated and empirical FCs. Two methods of pruning structure are taken: (1) SC is pruned based on absolute strength (the value below a certain strength would be omitted from the structural connectivity), ranging from the value between 0.02 to 0.08. (2) SC is pruned by percentile threshold, where a certain percentile of stronger edges or connections are kept, and others are omitted (set to zero). Four values of percentile threshold parameters (5, 10, 15, and 20 percentile) are selected, and only the connections that lie above a certain threshold are kept intact. How the structure changes with perturbation is presented in terms of the degree distribution in Fig. S4, along with the virtual view in Fig. S5 in the online supporting document.

For rehabilitating the FC from structural aberrations, both the Global coupling factor (G) and the oscillation amplitude $\mu$ are used to restore the FC after the structural pruning process. Global coupling factor (G) is altered from 10 to 40 (in case of without perturbation, it was set at 1), and the oscillation amplitude $\mu$ is increased from 2 to 4, where all the nodes are homogeneous in nature. Prior works also show that both parameters are important for restoring the aberration of the resting state FC incurred due to structural loss^11,14,21,45^. Note that for the resting state, the Hopf oscillators are assumed in a self-excitatory periodic oscillatory phase where the amplitude of the oscillation is defined by $\mu$ as given in equation 2. Similarly, increased G represents the increased structural connection, increased conduction velocity, and also lowering of it emulates the diminution of plasticity, which was explored earlier^38,45,61^.

### Supplementary Information

Supplementary Information.

## Data Availability

The datasets used and/or analysed during the current study are available in the Figshare online repository, https://figshare.com/articles/dataset/Paris_HCP_brain_connectivity_data/3749595/1.
