# Supplementary material for: A phenomenological model of whole brain dynamics using a network of neural oscillators with power-coupling
Source: Sci Rep. 2023 Oct 7;13:16935. doi: 10.1038/s41598-023-43547-3 (PMC10560247; doi:10.1038/s41598-023-43547-3)
Supplement: Supplementary file 1 — Supplementary Information. [file 41598_2023_43547_MOESM1_ESM.pdf]

A Phenomenological model of Whole Brain Dynamics Using a Network of Neural Oscillators with Power-Coupling

Anirban Bandyopadhyay[1], Sayan Ghosh[1], Dipayan Biswas[1], V. Srinivasa Chakravarthy[1] , and Bapi Raju S[2]

1 Indian Institue of Technology Madras, Biotechnology, Chennai, 600036, India

2 IIIT Hyderabad, Biotechnology, Hyderabad, 500008, India

S1. Description of Model architecture: -

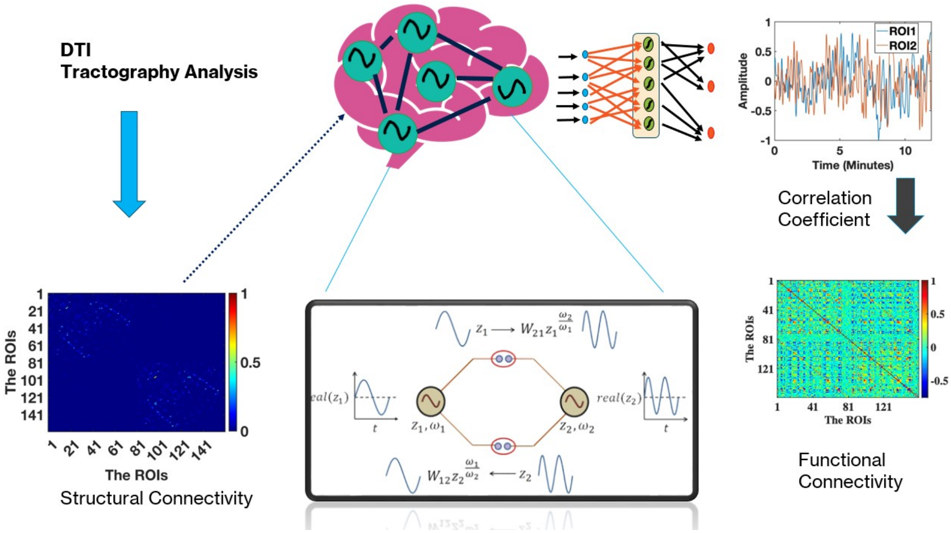

Figure S1. The schematic description of the model. The diagram shows that the brain is modeled as a network of Hopf oscillators whose connections are constrained by the empirical SC data obtained from DTI, and nature of connections is characterized by "power coupling". The Structural Connectivity and Functional Connectivity is taken from current work. Depiction of power-coupling is taken form earlier work[1].

A. The first phase of learning: -

As described earlier the network of N number of Hopf oscillators is used to learn a signal (i.e., teaching signal), the Network dynamics is as follows: -

$$\dot{Z}_\iota = Z_i(\mu + i\omega_i - |Z|^2) + G \sum_{j=1, j\neq i}^{N} A_{ij}\, e^{\frac{i\theta_{ij}}{\omega_j}} Z_j^{\frac{\omega_i}{\omega_j}} + \varepsilon e(t) \ldots\ldots\ldots\ldots\ldots\ldots\ldots\ldots..(1)$$

The error e(t) is calculated from the equation 6. For the lateral connection learning, the connections are partly set using the empirical. But the phase is trained following the Hebbian plasticity rule as shown in equation (4).

$$\dot{\omega}_\iota = -\beta_w e(t) \sin\varphi \ldots\ldots\ldots\ldots\ldots\ldots\ldots\ldots\ldots\ldots\ldots\ldots\ldots\ldots\ldots\ldots\ldots\ldots\ldots\ldots\ldots\ldots\ldots (2)$$

$$W_{ij} = A_{ij} e^{\frac{i\theta_{ij}}{\omega_j}} \ldots\ldots\ldots\ldots\ldots\ldots\ldots\ldots\ldots\ldots\ldots\ldots\ldots\ldots\ldots\ldots\ldots\ldots\ldots\ldots\ldots\ldots\ldots. (3)$$

$$\tau_w \dot{W}_{\iota J} = -W_{ij} + Z_i Z_j^{*\frac{\omega_i}{\omega_j}} \ldots\ldots\ldots\ldots\ldots\ldots\ldots\ldots\ldots\ldots\ldots\ldots\ldots\ldots\ldots\ldots\ldots\ldots\ldots\ldots (4)$$

$$p(t) = \sum_{i=1}^{N} \alpha_i cos\varphi_i \ldots\ldots\ldots\ldots\ldots\ldots\ldots\ldots\ldots\ldots\ldots\ldots\ldots\ldots\ldots\ldots\ldots\ldots\ldots\ldots\ldots\ldots..(5)$$

$$e(t) = D(t) - p(t) \ldots\ldots\ldots\ldots\ldots\ldots\ldots\ldots\ldots\ldots\ldots\ldots\ldots\ldots\ldots\ldots\ldots\ldots\ldots\ldots\ldots\ldots\ldots (6)$$

The learning parameters, β_w, and τ_w are set as 10^{-4} , and 10^4 for the learning rule for respectively the frequency learning, forward weight connection, and the Hebbian weight learning.

B. The second phase of learning: -

A succinct description of this deep oscillatory neural network is discussed in this section. Governing equation for the feedforward network is given below —

Least square error function/cost function with respect to each ROI has been taken here.

$$E = \frac{1}{2}\left(\sum_{i=0}^{N} (Y_{d,i} - Y_{p,i})^2\right) \ldots\ldots\ldots\ldots\ldots\ldots\ldots\ldots\ldots\ldots\ldots\ldots\ldots\ldots\ldots\ldots\ldots\ldots\ldots\ldots. (7)$$

where, Y_d is the empirical signal, and Y_p is the simulated one.

For forward propagation,

$$Lhbs = W1 \otimes dim \ldots\ldots\ldots\ldots\ldots\ldots\ldots\ldots\ldots\ldots\ldots\ldots\ldots\ldots\ldots\ldots\ldots\ldots\ldots\ldots\ldots\ldots\ldots\ldots\ldots\ldots. (8)$$

where, W1 be the weight matrix and the dim represent the simulated output which is complex in nature, from a collection of Hopf oscillators associated with each ROI
each ROI's BOLD signal.

The activation function used in this work is a special type of activation function, defined by-

$$2sigmf(X,\ a_k, c_k) - 1 \ldots\ldots\ldots\ldots\ldots\ldots\ldots\ldots\ldots\ldots\ldots\ldots\ldots\ldots\ldots\ldots\ldots\ldots\ldots\ldots\ldots\ldots(9)$$

Where, "simgf" refers to sigmoidal membership function represented by

$$\text{Sigmf}(X,a_k,c_k)= \frac{1}{1+e^{-a_k(X-c_k)}}$$

where, a_k= 0.5, and c_k=0.

For hidden layer to output layer,

$$Shbsr = \sigma\big(Real(Lhbs)\big); Shbsi = \sigma\big(imag(Lhbs)\big); Shbs = Shbsr + shbsi \ldots\ldots. (10)$$

$$sobs = W2 \otimes shbs \ldots\ldots\ldots\ldots\ldots\ldots\ldots\ldots\ldots\ldots\ldots\ldots\ldots\ldots\ldots\ldots\ldots\ldots\ldots\ldots\ldots\ldots\ldots\ldots\ldots\ldots. (11)$$

Only the real output is taken for the simulation, the imaginary output is disregarded. So,

$$Yp = \sigma\big(Real(Sobs)\big) \ldots\ldots\ldots\ldots\ldots\ldots\ldots\ldots\ldots\ldots\ldots\ldots\ldots\ldots\ldots\ldots\ldots\ldots\ldots\ldots\ldots\ldots\ldots\ldots\ldots (12)$$

The complex backpropagation rule for the network training is based on gradient descent, where the real part and the complex part of the weights are updated individually. The backpropagation rule closely follows an earlier work[2]. The back propagation rule is as followed-

The back-propagation rule is as follows-

$$W2_{real} = W2_{real} - \eta_{w2r} \frac{\delta E}{\delta W2_{real}} \ldots\ldots\ldots\ldots\ldots\ldots\ldots\ldots\ldots\ldots\ldots\ldots\ldots\ldots\ldots\ldots\ldots\ldots\ldots \quad (13)$$

$$W2_{imag} = W2_{imag} - \eta_{w2i} \frac{\delta E}{\delta W2_{imag}} \ldots\ldots\ldots\ldots\ldots\ldots\ldots\ldots\ldots\ldots\ldots\ldots\ldots\ldots\ldots\ldots\ldots\ldots \quad (14)$$

$$W2 = W2_{real} + iW2_{imag} \ldots\ldots\ldots\ldots\ldots\ldots\ldots\ldots\ldots\ldots\ldots\ldots\ldots\ldots\ldots\ldots\ldots\ldots\ldots\ldots \quad (15)$$

$$W1_{real} = W1_{real} - \eta_{w1r} \frac{\delta E}{\delta W1_{real}} \ldots\ldots\ldots\ldots\ldots\ldots\ldots\ldots\ldots\ldots\ldots\ldots\ldots\ldots\ldots\ldots\ldots\ldots\ldots \quad (16)$$

$$W1_{imag} = W1_{imag} - \eta_{w2i} \frac{\delta E}{\delta W1_{imag}} \ldots\ldots\ldots\ldots\ldots\ldots\ldots\ldots\ldots\ldots\ldots\ldots\ldots\ldots\ldots\ldots\ldots\ldots \quad (17)$$

$$W1 = W1_{real} + iW1_{imag} \ldots\ldots\ldots\ldots\ldots\ldots\ldots\ldots\ldots\ldots\ldots\ldots\ldots\ldots\ldots\ldots\ldots\ldots\ldots\ldots \quad (18)$$

In each iteration/epoch, the complex weights are updated, and the learning rate is defined by η. Different η values are given in the Table S1 below.

Parameter Table:-

Table S1. Definitions and values of parameters used in this study. [Kindly note that, without perturbation condition or in case of the basic model the μ, and G are kept at 1.]

Parameter	Definition	Values
μ	Bifurcation Parameter	$\mu \in \mathbb{R} \mid 1 \leq \mu \leq 40$ [μ values for perturbation cases are also included]
G	Global Bifurcation parameter	$G \in \mathbb{R} \mid 1 \leq G \leq 120$ [G values for perturbation cases are also included]
β_w	Learning parameter of frequency learning	10^{-4}
τ_w	Learning parameter of Hebbian learning rule	10^4
η_{w2r}, η_{w2i}, η_{w1r}, η_{w1i},	Learning parameters for back propagation	0.001
a_k	Parameter for sigmoid membership function	0.5
c_k	Parameter for sigmoid membership function	0

S2. Table of all parcellated brain region: -

Table S2. List of the Brain regions in left hemisphere. [For right hemisphere it follows same pattern of index] [3].

lG_and_S_frontomargin	lG_oc.temp_lat.fusifor	lLat_Fis.post	lS_oc.temp_med_and_Lingual
lG_and_S_occipital_inf	lG_oc.temp_med.Lingual	lPole_occipital	lS_orbital_lateral
lG_and_S_paracentral	lG_oc.temp_med.Parahip	lPole_temporal	lS_orbital_med.olfact
lG_and_S_subcentral	lG_orbital	lS_calcarine	lS_orbital.H_Shaped
lG_and_S_transv_frontopol	lG_pariet_inf.Angular	lS_central	lS_parieto_occipital
lG_and_S_cingul.Ant	lG_pariet_inf.Supramar	lS_cingul.Marginalis	lS_pericallosal
lG_and_S_cingul.Mid.Ant	lG_parietal_sup	lS_circular_insula_ant	lS_postcentral
lG_and_S_cingul.Mid.Post	lG_postcentral	lS_circular_insula_inf	lS_precentral.inf.part
lG_cingul.Post.dorsal	lG_precentral	lS_circular_insula_sup	lS_precentral.sup.part
lG_cingul.Post.ventral	lG_precuneus	lS_collat_transv_ant	lS_suborbital
lG_cuneus	lG_rectus	lS_collat_transv_post	lS_subparietal
lG_front_inf.Opercular	lG_subcallosal	lS_front_inf	lS_temporal_inf
lG_front_inf.Orbital	lG_temp_sup.G_T_transv	lS_front_middle	lS_temporal_sup
lG_front_inf.Triangul	lG_temp_sup.Lateral	lS_front_sup	lS_temporal_transverse
lG_front_middle	lG_temp_sup.Plan_polar	lS_interm_prim.Jensen	Thalamus
lG_front_sup	lG_temp_sup.Plan_tempo	lS_intrapariet_and_P_trans	Caudate
lG_Ins_lg_and_S_cent_ins	lG_temporal_inf	lS_oc_middle_and_Lunatus	Putamen
lG_insular_short	lG_temporal_middle	lS_oc_sup_and_transversal	pallidum
lG_occipital_middle	lLat_Fis.ant.Horizont	lS_occipital_ant	Hippocampus
lG_occipital_sup	lLat_Fis.ant.Vertical	lS_oc.temp_lat	Amygdala

S3. Identifying the "Brain state" during FCD calculation-

The methodology to deduce the FCD is based on Menon et al [4], which offers us a chance to compare the "Brain state" between simulated, and empirical BOLD signal. It can be obtained by sliding window analysis (SWA) resulting in a series of FC matrices, where K-means clustering (K=4) is employed to segregate the FC matrices into four clusters, and the centroid of each cluster is called a "brain state". Note that, due to applying MATLAB's K means clustering algorithm the numbering assignment or indexing to the states is different in each iteration of the program.

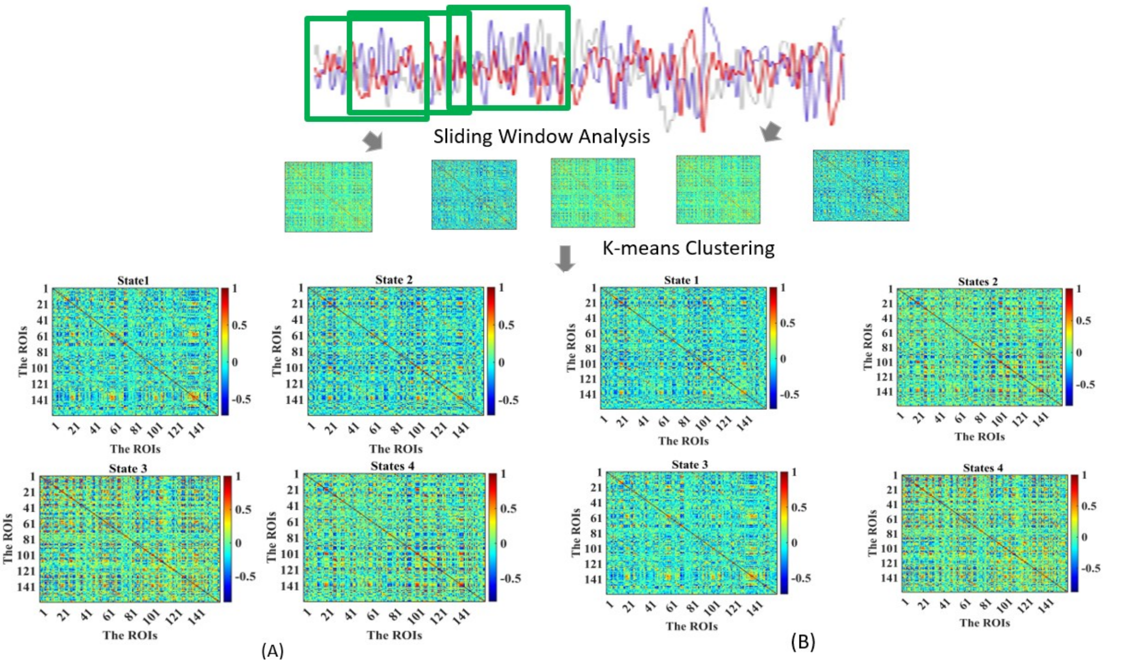

Figure S2. In panel (A) four FCs are obtained from the empirical BOLD signals, while the other four FCs shown in panel (B) are from the simulated outcome.

.

S4. Graph Theory and Community Affiliation vector: -

"Brain Connectivity toolbox" (BCT) provides the opportunity to opt for revised Newman's clustering algorithm for community analysis. Here the nodes associated with community affiliation vector for each simulated, and empirical FC are noted [5]. Pursuing classical graph theory, the modularity value of the surrogate data is also noted. For this, Fourier's transformation is performed, where the phase sequence is randomized and then retrieved with the inverse Fourier transformation to get the surrogate empirical data [6].

The below result is for only first participants' data from HCP dataset, and similar kind of result is observed from Paris dataset. The test such as FC and FCD estimation, and default mode network identification are done to pass the validation tests set by Cabral et al. for large-scale brain models[7].

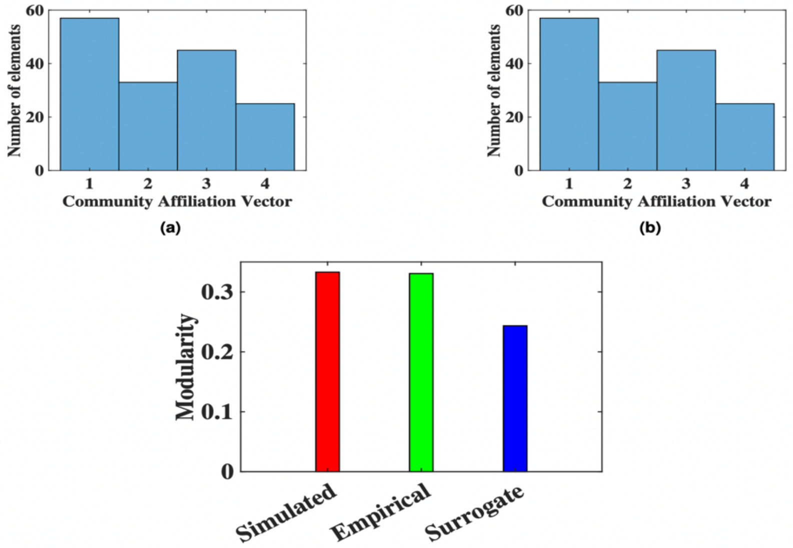

Figure S3. (a) Number of ROI in the respective community for experimental data; (b) Number of ROIs in respective community for simulated data; (c) the modularity value for empirical data, simulated data, and surrogate data for human connectome project dataset.

It is to be noted that there is no consensus regarding which is the best methodology to deduce the graph theoretical measures from FC matrices. Here "grand-average" or group-average of FC matrices are taken after the model's simulated FC matrices are obtained, and this "grand-average" FC matrix is used to deduce the community identification. This methodology is congruent with the earlier work published in literature domain aimed to calculate modularity[8,9,10].

However, in this process, there is a high chance that we may miss to account for the inter-subject variation. To address this problem, a ratio between mean to standard deviation of FC

matrices for individual participants can be taken, which is defined by Chen et al[11]. This looks like as follows-

$$n_{i,j} = \frac{\frac{1}{N}\sum_{m=1}^{N} a_{m,i,j}}{\sqrt{\frac{1}{N}\sum_{m=1}^{N}\left(a_{m,i,j} - \mu_{i,j}\right)^2}}$$

Where, $a_{m,i,j}$ reveals the correlation coefficient value, N represents the total number of subjects, $n_{i,j}$ is the correlation coefficient value for the representative matrix of a group. Also, only positive correlation value has been taken into account for this process, the negative ones are set to zero. However, our results point out that there is no significant alteration in modularity analysis and community detection. Similar to the current analysis, four modular structures are found with the representative matrix as well. DMN regions are located within one of the communities.

S5. Structural loss and Rehabilitation

In the main text, we present how structural loss (similar to random attack in graph theory), can cause aberration in functional connectivity, and functional connectivity dynamics. The FC (correlation between entire time-series signal for both simulated, and empirical) and FCD in terms of correlation between FC at each time -window, and correlation with it with all other FC for all other existing time-window calculated with sliding window analysis technique is reported. Here, FCD matrix is different from the one which is shown in the main text. It is a N×N matrix, where each element represents the correlation coefficient between two FC matrices rendered by the sliding window analysis.

At first, we have shown the impact of structural lesion on the degree of brain-graph; and a virtual view of it is given in Figs. S4, and S5. After that, we have given all the perturbation cases, and how the simulated FC and FCD are impacted by it. At the end, a histogram is given for depicting the dynamic correlation for each perturbation case. All results shown below are for single participant's data.

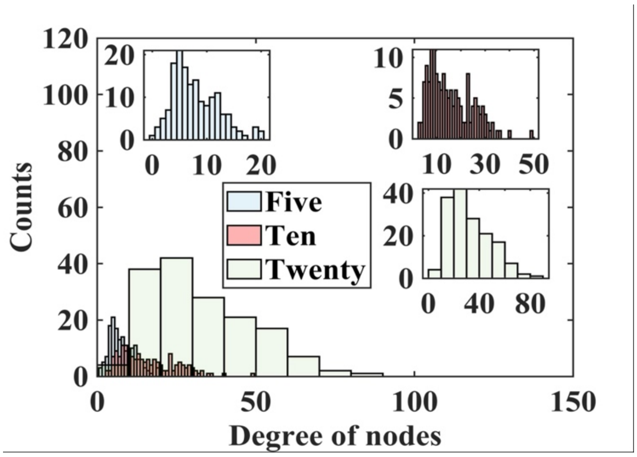

Figure S4. Histogram plot for degree distribution of the nodes of structural connectivity. It refers how many nodes have certain degree or certain number of neighbors after the pruning process. As the multi-histogram plot gets a little ambiguous, in the inset, the degree distribution for the nodes where five, ten, and twenty percentile threshold applied on structural connectivity.

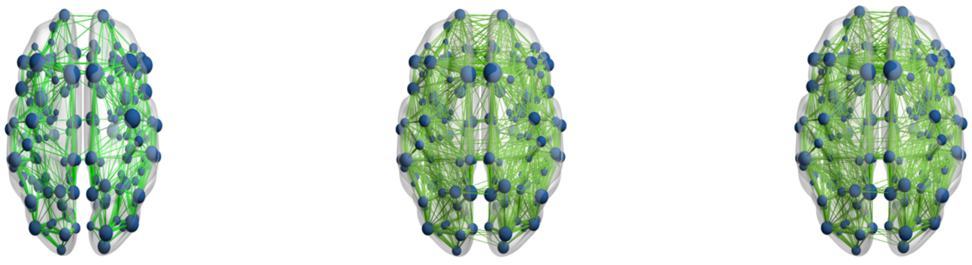

(A) (B) (C)

Figure S5. Structural Connectivity from axial view [structural connectivity with only five, ten, and twenty percentile thresholds.]

A. <u>FC and FCD without pruning: -</u>

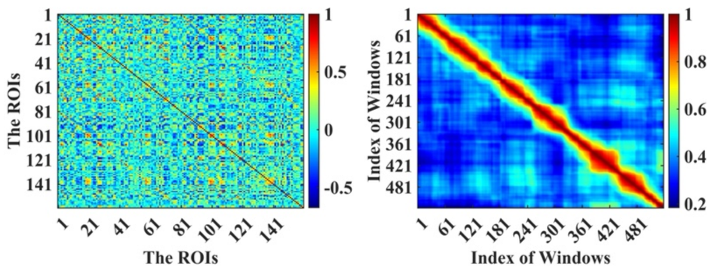

(A) (B)

Figure S6. The two symmetric matrices, individually, show the simulated Functional connectivity and Functional connectivity dynamics, when no pruning is done on the structural connectivity matrix. The result is shown here for the first participant indexed in the HCP dataset.

B. FC and FCD with proportional thresholding:-

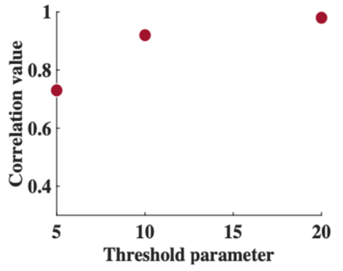

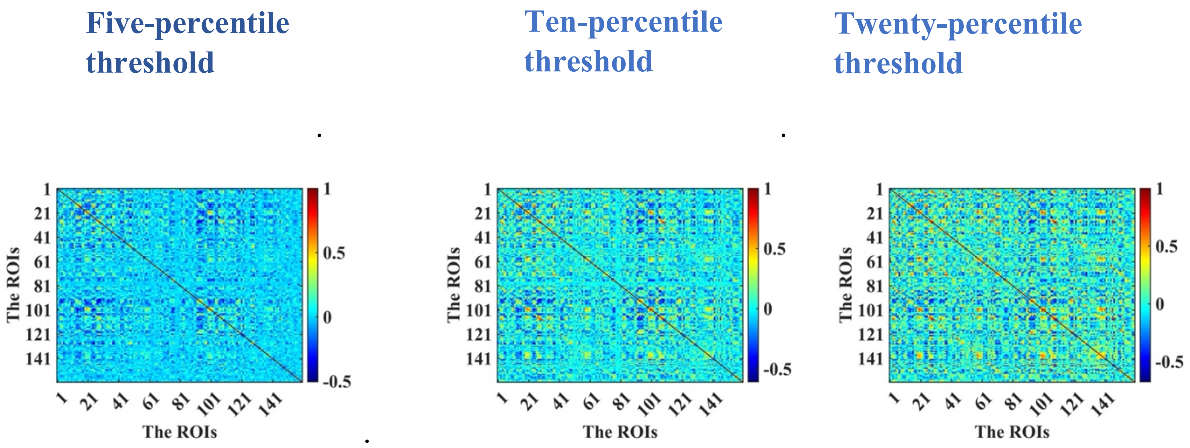

(A)

Functional Connectivity

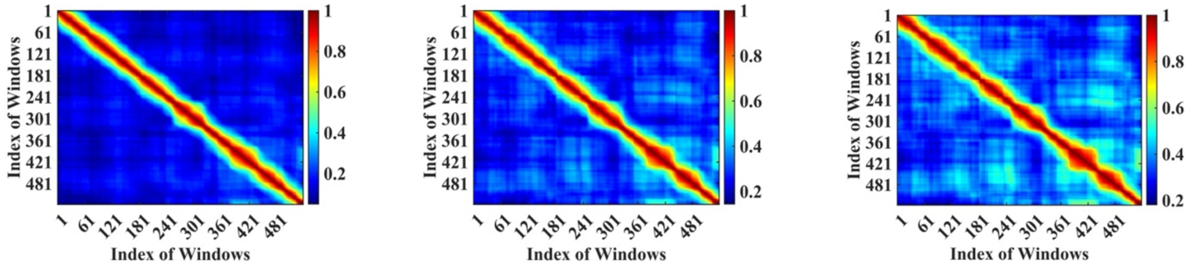

(B)

Functional Connectivity Dynamics

Figure S7. This represents the functional connectivity, and functional connectivity dynamics for structural connectivity with different thresholds. It is the extension of the result shown in Fig. 3 (b). It is evident, how the FC and FCD are dependent on the threshold parameter.

C. FC and FCD with five percentile threshold and increased G: -

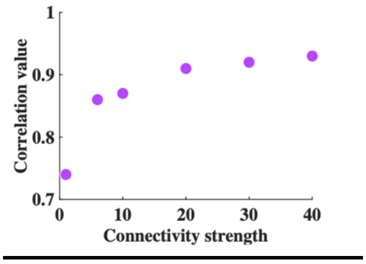

G= 1, five percentile threshold

G= 20, five percentile threshold

G= 40, five percentile threshold

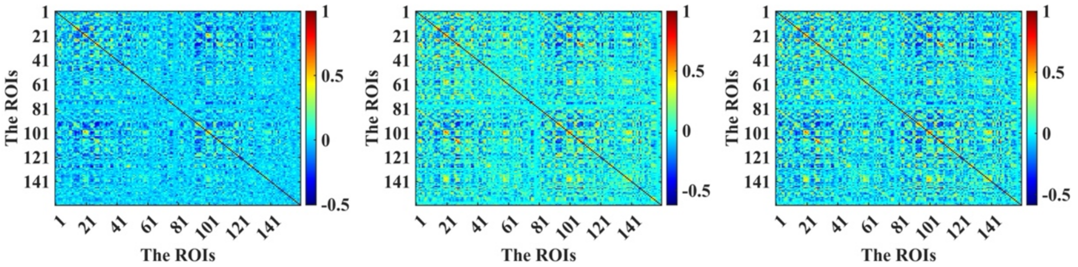

(A)

Functional Connectivity

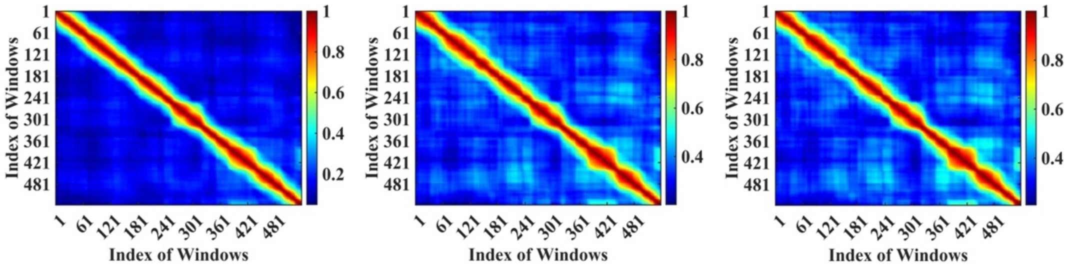

(B)

Functional Connectivity Dynamics

Figure S8. Figs. (A) and (B) represent the FC and FCD dynamics when the global coupling factor (G) is increased from G=1 to G=40, when the structural connectivity is pruned in a way so that five percentile strong connection remains. It can be visualized that the increase in G almost compensates for the structural loss in structural connectivity matric, and reflects in FC, and FCD. FC and FCD at G=40 almost resembles the empirical FC and FCD.

D. <u>FC and FCD with five percentile threshold with increased bifurcation parameter: -</u>

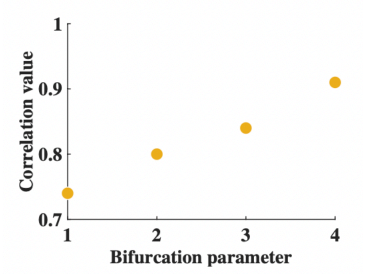

μ= 1, five percentile threshold **μ= 2, five percentile threshold** **μ= 4, five percentile threshold**

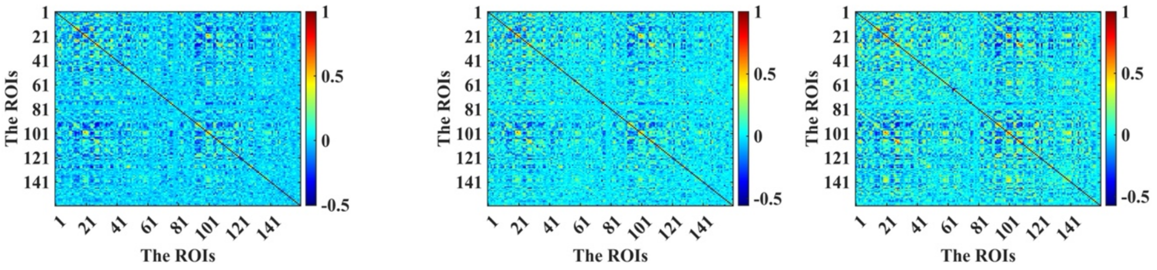

(A)

Functional Connectivity

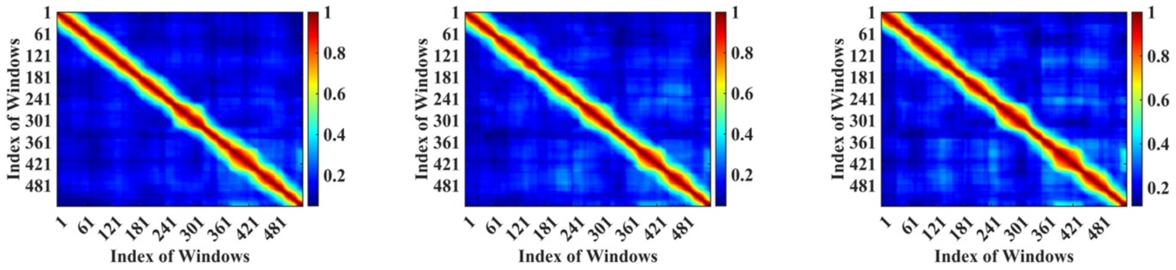

(B)

Functional Connectivity Dynamics

Figure S.9. Figs. (A) and (B) represent the FC and FCD dynamics when the oscillation amplitude of the Hopf-oscillation is set to increase from 2 to 4, when the structural connectivity is artificially lesioned, and only five percentile strongest connection remains. It can be visualized that the increase in oscillation amplitude almost compensates for the structural loss in the structural connectivity matrix, and reflects in FC and FCD. FC and FCD at bifurcation parameter, μ=4 almost resemble the empirical FC and FCD. This result substantiates the outcome prescribed in Figs. 3. (e) and 4. (c) in text.

E. FC and FCD with ten percentile threshold with increased G: -

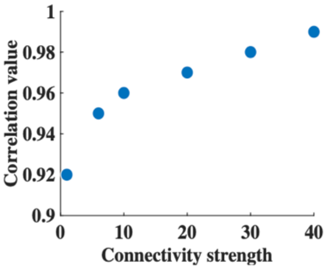

G= 1, ten percentile threshold

G= 20, ten percentile threshold

G= 40, ten percentile threshold

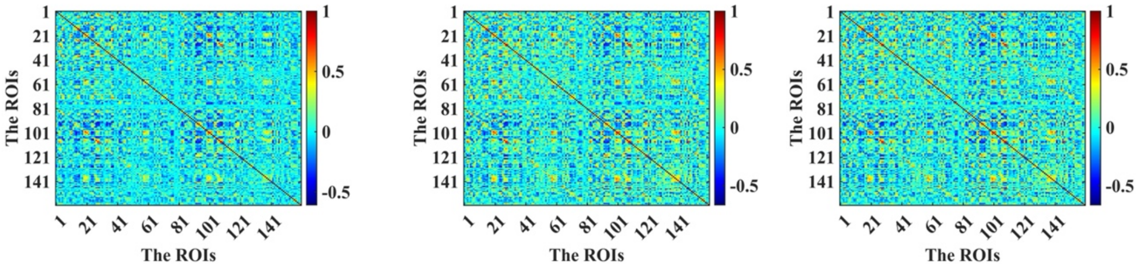

(A)

Functional Connectivity

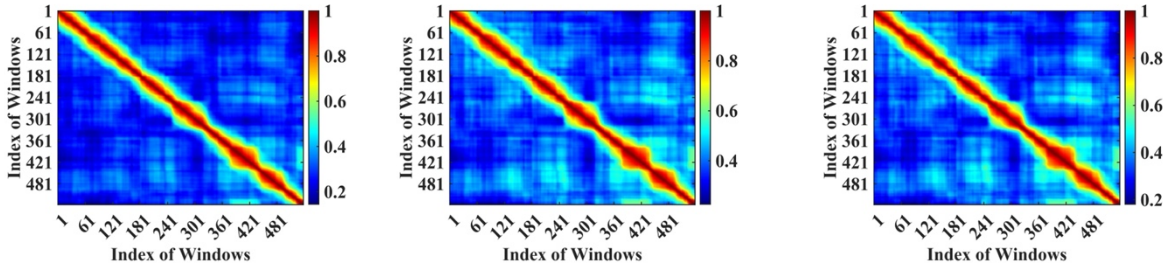

(B)

Functional Connectivity Dynamics

Figure S10. Figs. (A) and (B) represent the FC and FCD dynamics, when the global coupling factor (G) is increased from G=1 to G=40, and when only the ten percentile strongest connection remains. It can be visualized that the increase in G almost compensates for the structural loss in the structural connectivity matrix, and reflects in FC and FCD. Simulated FC and FCD, when G=40, almost resembles the empirical FC and FCD.

F. FC and FCD with ten percentile thresholds, and increased bifurcation parameter: -

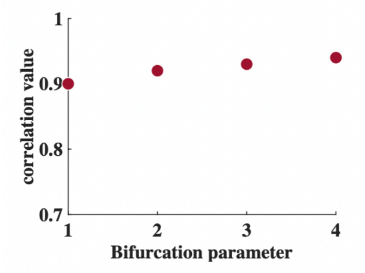

μ= 1, ten percentile threshold

μ= 2, ten percentile threshold

μ= 4, ten percentile threshold

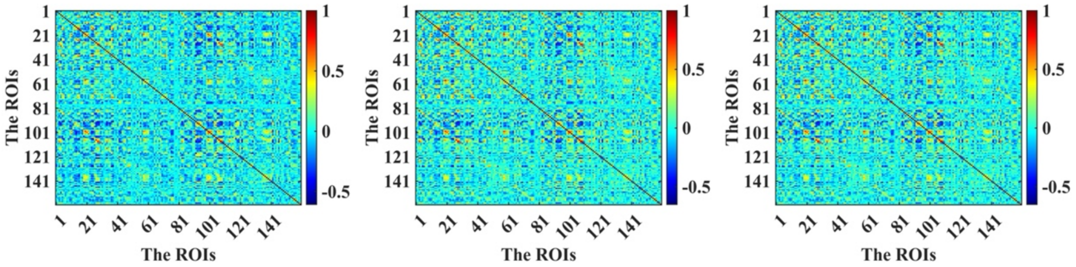

(A)

Functional Connectivity

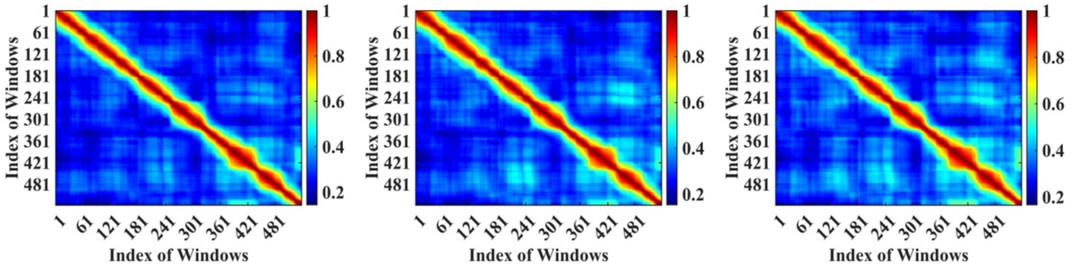

(B)

Functional Connectivity Dynamics

Figure S11. Figs. (A) and (B) represent the FC and FCD dynamics when the amplitude of the Hopf-oscillation is set to increase from 1 to 4, when the structural connectivity is artificially lesioned, and only ten percentile strongest connection remains. It can be visualized that the increase in bifurcation parameter almost compensates for the structural loss in structural connectivity matric, and reflects in FC and FCD. FC, and FCD at bifurcation parameter, μ =4 almost resembles the empirical FC, and FCD. This result substantiates the outcome prescribed in Figs. 3. (f) and 4. (e) in text. However, as correlation value is already at 0.91 for bifurcation parameter 1; that's why it is cumbersome to distinguish between them easily.

G. <u>The distribution of the dynamic correlation: -</u>

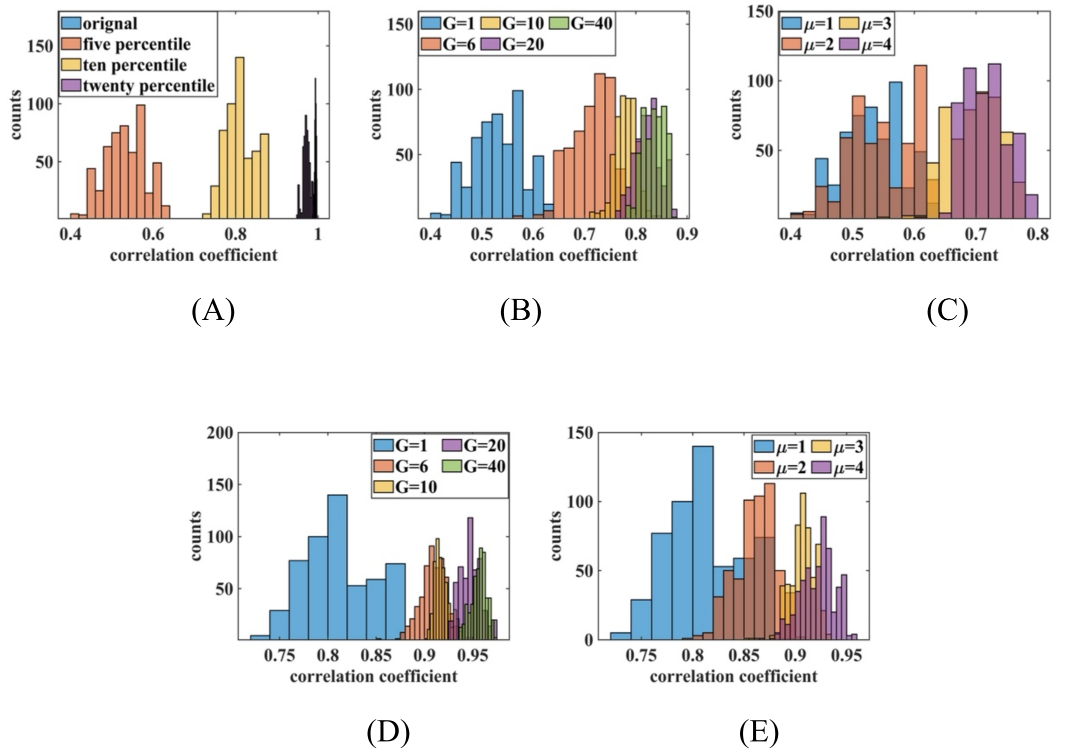

Figure S12. The distribution of dynamic correlation as shown in Fig. 4. in the original text. Fig. (A) represents the distribution of dynamic correlation for five, ten and twenty percentile thresholds for structural connectivity. The 'original' represents when no threshold parameter is applied for structural connectivity. Fig. (B) refers to the dynamic correlation distribution for different global coupling factors (G), when only five percentile strongest connection is present. Fig. (C) refers to the dynamic correlation distribution for different bifurcation parameters, when only five percentile strongest connection is present. Fig. (D) refers to the dynamic correlation distribution for different global coupling factors (G), when only ten percentile strongest connection is present. Fig. (E) refers to the dynamic correlation distribution for different bifurcation parameters, when only ten percentiles of strongest connection are present.

H. <u>Limiting Cases:-</u>

In this section, we will discuss the limiting cases where the FC and FCD cannot be restored after the pruning process. After further investigation into the pruning procedure, it is noticed that the governing parameters of the coupled Hopf-oscillator-based dynamical systems, like the bifurcation parameter, μ and global coupling factor, G, cannot increase the correlation coefficient when the proportional threshold is kept at low percentile threshold value, where only few strongest connections are present. For example, a study is done when only one percentile (much less than five percentile) strongest connections are kept intact, and others are set to zero. It reveals that the correlation coefficient value between simulated and empirical FC

matrices comes down to 0.12. It shows that extreme pruning of the SC matrix causes severe damage in the FC matrix. Optimization of the correlation coefficient is done with both G and μ individually with a wide range of values in order to restore the original correlation coefficient value which is simulated without pruning procedure, and it is 0.98. However, it is found that the correlation coefficient value does not increase linearly with G and μ after a certain point. Perturbation is done both with increased G and μ, the correlation coefficient values attain saturation, which is reflected by both FC analysis and FCD analysis. Fig. S13. (A) and (B) show the pattern of increase in correlation coefficient, and FCD analysis is shown for different perturbation-condition with G and μ in terms of D_{ks} or Kolmogorov-Smirnov distance similar to the analysis given in Fig. 4. (f) in the main text. D_{ks} value is also not changing linearly with increased G and μ as shown in Fig. S13. (C). This study is just a preliminary work showcasing the characteristics of the model. Detailed work on such in silico perturbation study will be done in our future work to demystify the impact of FC due to structural aberration in cases of neurological disorders.

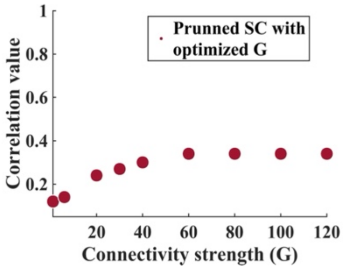

(A)

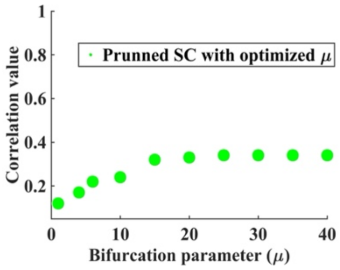

(B)

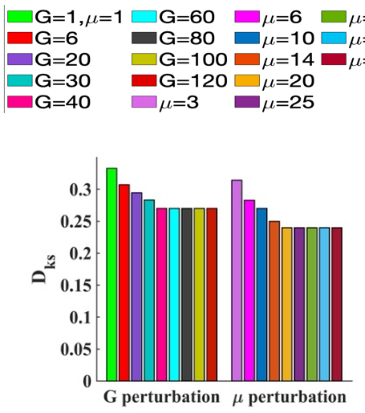

(C)

Figure S13. FC, and FCD analysis for different perturbation condition after one-percentile threshold. Fig. (A), and (B) show the FC analysis, where the first point refers to the estimated correlation coefficient, when both G, and μ is kept at 1. Fig. (C) shows the FCD analysis in terms of $D_{ks,}$ in both cases of G, and μ perturbation. First value (marked with light green) represents the $D_{ks,}$ value, when both G, and μ is kept at 1.

References:-

1. Biswas, D., Pallikkulath, S. & Chakravarthy, V. S. A complex-valued oscillatory neural network for storage and retrieval of multidimensional aperiodic signals. *Front. computational neuroscience* 15, 551111 (2021).
2. Georgiou, G. M. & Koutsougeras, C. Complex domain backpropagation. *IEEE transactions on Circuits systems II: analog digital signal processing* 39, 330–334 (1992).
3. Marrelec, G., Messé, A., Giron, A. & Rudrauf, D. Functional connectivity's degenerate view of brain computation;figshare*:* https://figshare.com/articles/dataset/Paris_HCP_brain_connectivity_data/3749595/1 (2016).
4. Menon, S. S. & Krishnamurthy, K. A comparison of static and dynamic functional connectivities for identifying subjects and biological sex using intrinsic individual brain connectivity. *Sci. reports* 9, 1–11 (2019).
5. Rubinov, M. & Sporns, O. Complex network measures of brain connectivity: uses and interpretations. *Neuroimage* 52, 1059–1069 (2010).
6. Lancaster, Gemma, Dmytro Iatsenko, Aleksandra Pidde, Valentina Ticcinelli, and Aneta Stefanovska. "Surrogate data for hypothesis testing of physical systems." *Physics Reports* 748 (2018): 1-60.
7. Cabral, Joana, Morten L. Kringelbach, and Gustavo Deco. "Functional connectivity dynamically evolves on multiple time-scales over a static structural connectome: Models and mechanisms." *NeuroImage* 160 (2017): 84-96.
8. Bordier, Cécile, Carlo Nicolini, and Angelo Bifone. "Graph analysis and modularity of brain functional connectivity networks: searching for the optimal threshold." *Frontiers in neuroscience* 11 (2017): 441.
9. Rudie, Jeffrey D., J. A. Brown, Devi Beck-Pancer, L. M. Hernandez, E. L. Dennis, P. M. Thompson, S. Y. Bookheimer, and M. J. N. C. Dapretto. "Altered functional and structural brain network organization in autism." *NeuroImage: clinical* 2 (2013): 79-94.
10. Hahn, Gerald, Gorka Zamora-López, Lynn Uhrig, Enzo Tagliazucchi, Helmut Laufs, Dante Mantini, Morten L. Kringelbach, Bechir Jarraya, and Gustavo Deco. "Signature of consciousness in brain-wide synchronization patterns of monkey and human fMRI signals." *NeuroImage* 226 (2021): 117470.
11. Chen, Guangyu, Hong-Ying Zhang, Chunming Xie, Gang Chen, Zhi-Jun Zhang, Gao-Jun Teng, and Shi-Jiang Li. "Modular reorganization of brain resting state networks and its independent validation in Alzheimer's disease patients." *Frontiers in human neuroscience* 7 (2013): 456.
